# Retrieval of fragmented peripherally inserted central catheter (PICC) with a double transfemoral access technique

**DOI:** 10.1002/ccr3.3189

**Published:** 2020-08-02

**Authors:** Alfonso Papa, Dario Tammaro, Vittorio Monda

**Affiliations:** ^1^ Pain Department AO Ospedali dei Colli Monaldi Hospital Napoli Italy; ^2^ CVC Team AO Ospedali dei Colli Monaldi Hospital Napoli Italy; ^3^ Division of Cardiology AO Ospedali dei Colli Monaldi Hospital Napoli Italy

**Keywords:** CVC fragment, peripherally inserted central catheter, retrieval CVC

## Abstract

Retrieval of central venous catheters fragments often puts us in front of different situations. Having more techniques available for strategic planning of the procedure is important. The authors propose the simultaneous use of two different approaches for the recovery of a CVC fragment from the pulmonary artery.

## Graphical abstract

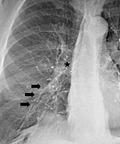

## 1 CASE DESCRIPTION

Retrieval of central venous catheter fragments often puts us in front of different situations. Having more techniques available for strategic planning of the procedure is important. The authors propose the simultaneous use of two different approaches for the recovery of a CVC fragment from the pulmonary artery.

**FIGURE 1:**
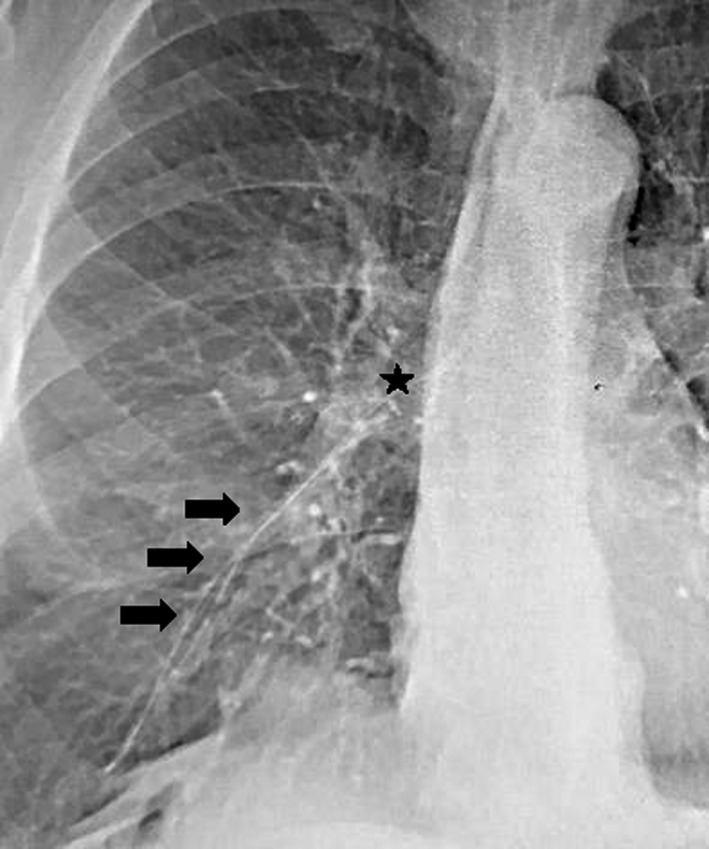
Fragmented PICC migrated into lower branch of right pulmonary artery (arrow) and medially positioned loop (star)

For vascular catheter fragment recovery, there are several techniques including use of pigtail catheter or gooseneck snare.^1^, ^2^ We describe the case of a PICC fragment migrated into lower branch of right pulmonary artery Figure 1Retrieval using a pigtail catheter and a gooseneck snare inserted at the same time with a double transfemoral access. The pigtail catheter was used to withdraw the catheter from the pulmonary artery, while at the same time, the gooseneck snare was used to grasp the end of fractured catheter to prevent blood flow from returning the fragment to the pulmonary artery Video S1.

## CONFLICTS OF INTEREST

The authors state that they have no conflicts of interest. This research did not receive any specific grant from funding agencies in the public, commercial, or not‐for‐profit sectors.

## AUTHOR CONTRIBUTIONS

AP: involved in conception and drafting of the article and video storing. DT and VM: performed technique and acquire images.

## ETHICAL STATEMENT

This study was in accordance with the 1964 Helsinki Declaration and its later amendments or comparable ethical standards.

## Supporting information

Video S1
